# Effectiveness of King’s Theory of Goal Attainment in Blood Glucose Management for Newly Diagnosed Patients With Type 2 Diabetes: Randomized Controlled Trial

**DOI:** 10.2196/59142

**Published:** 2024-10-31

**Authors:** Man Yan, Yingchun Yu, Shuping Li, Peiling Zhang, Jiaxiang Yu

**Affiliations:** 1 Changzhou Hospital of Traditional Chinese Medicine Changzhou China; 2 Department of Nursing and Rehabilitations Faculty of Medicine and Health Sciences University Putra Malaysia Serdang Malaysia

**Keywords:** King’s Theory of Goal Attainment, online feedback approach, newly diagnosed patients with type 2 diabetes mellitus, blood glucose control, type 2 diabetes, diabetes mellitus, blood glucose

## Abstract

**Background:**

Diabetes poses a significant public health challenge in China and globally, with the number of patients expected to reach 592 million by 2035, notably in Asia. In China alone, an estimated 140 million individuals are living with diabetes, and a significant portion is nonadherent to medications, underscoring the urgency of effective management strategies. Recognizing the necessity of early and comprehensive management for newly diagnosed patients with type 2 diabetes, this study leverages an online teach-back method and “Internet + Nursing” platform based on King’s Theory of Goal Attainment. The approach aims to enhance glycemic control and reduce fear and misconceptions about the disease, addressing both the educational and emotional needs of the patients.

**Objective:**

The primary aim of this study was to assess the effectiveness of King’s Goal Attainment Theory in the management of newly diagnosed patients with type 2 diabetes. This research sought to develop a collaborative model for blood glucose management, integrating the expertise and roles of physicians, nurses, and patients. The model is designed to enhance the synergy in health care provision, ensuring a comprehensive approach to diabetes management.

**Methods:**

In this study conducted at Changzhou Traditional Chinese Medicine Hospital between January 2022 and February 2023, eligible patients were randomized into a control group or an online feedback group. The control group received standard care, while the online feedback group participated in a King’s Theory of Goal Attainment–based online teach-back program, enhanced by “Internet + Nursing” strategies. This included an interactive platform for goal planning, video content sharing, comprehension assessment, misconception correction, and patient-driven recaps of disease information. Health monitoring was facilitated through the “Internet + Nursing” platform. The study focused on comparing changes in glucose metabolism and emotional disorder symptoms between the groups to evaluate the intervention’s effectiveness.

**Results:**

Following a 24-week intervention, we observed significant differences in key metrics between the online feedback group and the control group, each comprising 60 participants. The online feedback group demonstrated significant reductions in fasting plasma glucose, 2-hour postprandial glucose, and hemoglobin A_1c_ (*P*<.05). Additionally, there was a notable decrease in hypoglycemia-related anxiety and alexithymia within this group. Conversely, the control group maintained relatively higher values for these metrics at the same time point (*P*<.05). These findings underscore the efficacy of online feedback in managing glycemic control and reducing psychological distress associated with hypoglycemia.

**Conclusions:**

The online teaching-back method, guided by King’s Theory of Goal Attainment, effectively enhances glycemic control, reducing fasting plasma glucose, 2-hour postprandial glucose, and hemoglobin A_1c_ levels in newly diagnosed patients with type 2 diabetes. Simultaneously, it alleviates hypoglycemia-related anxiety and mitigates alexithymia. This approach merits widespread promotion and implementation in clinical settings.

**Trial Registration:**

Chinese Clinical Trial Registry ChiCTR2400079547; https://www.chictr.org.cn/showproj.html?proj=208223

## Introduction

Diabetes has emerged as a major challenge in China’s comprehensive prevention and management strategy, reflecting a global crisis with escalating prevalence worldwide [1,2]. The number of patients with diabetes is projected to rise to 592 million by 2035, with Asia, particularly China and India, at the epicenter of this pandemic. China currently leads with an estimated 140 million patients with diabetes [3,4]. Notably, a third of individuals with diabetes are nonadherent to prescribed medications. This highlights the severity and public health implications of diabetes management, now recognized as a critical global health issue.

Effective diabetes management is akin to a prolonged struggle [5,6]. There is a consensus among international scholars on the necessity of early, community-based, and multidimensional comprehensive management [7]. Particularly, patients with newly diagnosed type 2 diabetes often lack disease-specific knowledge and have misconceptions, leading to fear and inaccurate expectations about treatment outcomes.

At present, the multidisciplinary collaboration model has been widely recommended as an effective strategy for managing diabetes. Experts and scholars globally advocate for transforming diabetes care from traditional doctor-led approaches to multidisciplinary health care models. This transformation enables patients to work with professional health teams, including diabetes specialist nurses, psychological counselors, nutrition specialist nurses, and endocrinologists, to achieve optimal diabetes management through the implementation of healthy lifestyles and appropriate treatment interventions. Numerous studies have confirmed that personalized medical and nursing collaboration models can significantly improve patients’ quality of life and reduce risk factors [8-10].

To address these challenges, this study introduces an online teach-back method using a bidirectional information model, implementing a dynamic, stepwise approach for subtle health education [11]. Using the “Internet + Nursing” platform, we developed an intervention based on King’s Theory of Goal Attainment. This strategy aims to improve glycemic control and alleviate the fear of hypoglycemia and alexithymia in patients with newly diagnosed type 2 diabetes.

## Methods

### Study Population

This study involved first-onset patients with type 2 diabetes from Changzhou Traditional Chinese Medicine Hospital, recruited from January 2022 to February 2023, who met specific inclusion and exclusion criteria. Participants were randomly divided into a control group and an online feedback group using a random number table method. Inclusion criteria included (1) diagnosis of type 2 diabetes according to the 2017 Chinese Guidelines for the Prevention and Treatment of Diabetes; (2) ability to use basic mobile devices; (3) self-care capability, effective communication, and voluntary participation; and (4) access to a home blood glucose meter for self-monitoring. Exclusion criteria encompassed (1) acute diabetic complications or mental illnesses; (2) severe organ dysfunction; (3) malignant tumors; and (4) cognitive dysfunction, determined by Mini-Mental State Scale (MMSE) scores below 24 points. Additional exclusions during the study were due to personal dropout, withdrawal requests during follow-up, or unavailability for 2 consecutive follow-ups.

### Ethical Considerations

This study was conducted with the ethics approval of the Ethics Committee of Changzhou Hospital of Traditional Chinese Medicine, in strict accordance with the guidelines and regulations governing research involving human participants (2022-L-L-035(s)). Informed consent was obtained from all participants involved in the study. The confidentiality of all personal information and data collected during the study was assured.

### Control Group

Specialist diabetes nurses from the research team conducted comprehensive assessments of enrolled patients and established individual patient files. These files informed the development of personalized plans for diet, exercise, and glucose monitoring tailored to each patient’s general health information and blood sugar levels. Additionally, patients received individualized guidance plan record manuals and educational logs.

Routine health education was provided to all participants. This includes monthly patient education classes organized by the department, personalized educational sessions, distribution of diabetes-related materials, and regular follow-up visits as part of the ongoing care.

### Online Feedback Group

In addition to routine care, our hospital uses the “Internet + Nursing” platform to monitor patients’ disease status using a feedback approach. Using this platform, we established an interactive station that leverages the King’s standard theory for online teaching. This approach was further enhanced by problem-based learning techniques. We organize weekly group discussion activities, with the timing of these sessions coordinated and mutually agreed upon by team members. The research team was dedicated to managing and educating patients, providing timely responses to queries, conducting real-time supervision, and facilitating reverse retellings for patients.

### Management Process and Content

#### Informed Consent

Initially, we secured informed consent from the patient to ensure ethical standards and patient autonomy were upheld.

#### Comprehensive Assessment

We conducted a detailed questionnaire survey to understand the patient’s diabetes-related knowledge, self-management practices, lifestyle, and concerns about hypoglycemia. The purpose of this assessment was to gather comprehensive information to tailor the educational intervention accurately. The questionnaire was designed to capture critical data, which was then used in follow-up interviews to formulate a personalized goal-reaching plan. This approach helped to reduce the occurrence of bias and ensured that the final plan was well-suited to each patient’s needs.

#### Collaborative Goal Setting

We used a targeted questioning approach, such as the following:

Based on our established objectives, what areas do you need our assistance with for understanding? Which resources do you prefer for learning, e.g., videos or brochures?

This facilitated a mutual planning process to meet the patient’s goals, emphasizing cooperative efforts to achieve optimal glycemic control. The effectiveness of these tools was evaluated based on patient engagement and knowledge retention.

#### Educational Implementation

The educational strategy involved a structured process beginning with the distribution of video content, followed by organized review sessions, assessment of comprehension, clarification of misconceptions, and concluding with the patient’s recapitulation of disease-related information. This methodical approach ensured patients were actively engaged in their learning journey, promoting better health outcomes.

### Duration and Frequency

The intervention spanned 24 weeks, with weekly sessions lasting between 20 and 30 minutes each.

### Content and Structure

#### Initial Sessions (1-3)

It focused on imparting knowledge about diabetes, facilitated by diabetes specialist nurses and nutritionists.

#### Middle Sessions (4-9)

It covered self-management skills, including diet, medication, self-monitoring, and safety practices.

#### Psychological Support (10-12)

It was conducted by a senior psychotherapist, these sessions address the patient’s psychological well-being.

#### Doubt Clearance (13-16)

Doubt clearance had a diabetes specialist nurse engaged in question-and-answer sessions to resolve any patient queries.

#### Personalized Support (17-20)

Further individualized attention was given to strengthen knowledge and self-management skills.

#### Review and Summary (21-24)

Final sessions focus on answering outstanding questions and retrospective summarization. The intervention uses a hybrid of online one-on-one and group guidance for the initial 20 sessions. Specific strategies included the following.

#### Explanation

Using platforms like WeChat (Tencent Holdings Limited) for sharing video content, ensuring explanations were in layman’s terms to enhance comprehension.

#### Assessment

The teach-back method was used by asking patients or family members to paraphrase educational content and demonstrate learned techniques, assess the patient’s understanding of diabetes care–related knowledge and the accuracy of the blood sugar measurement process. For example, adopt an approachable attitude when asking the patient or family members questions like, “After attending this education session, can you tell me which fruits are safe to eat and which beverages should be avoided?” or “How and when do you usually measure your blood sugar?”

#### Clarification

Responsive feedback was provided, with misunderstandings addressed through further education and demonstration, ensuring clarity and accurate knowledge transfer.

#### Understanding

Open-ended questions facilitated the evaluation of comprehensive understanding and signaled the conclusion of the educational round when patients could independently recount the educational content.

### Observation Indicators

#### Laboratory Biochemical Indicators

Blood samples were collected to assess the efficacy of the intervention on glucose metabolism. Specifically, 3 mL of venous blood was drawn from patients 1 day before the intervention and another 3 mL of fasting venous blood the following morning postintervention. Samples were centrifuged to separate serum, which was then stored at –20 °C until analysis. Key glucose metabolism markers analyzed included fasting blood glucose (FBG), 2-hour postprandial glucose (2hPG), and hemoglobin A_1c_ (HbA_1c_).

#### Chinese Version of the Hypoglycemia Fear Survey II

The Chinese Version of the Hypoglycemia Fear Survey II (CHFS-II), adapted for Chinese populations by Mu Chun et al [12], is based on the original Hypoglycemia Fear Survey II. This version consists of the Chinese Hypoglycemia Fear-Behavior Scale (CHFS-II-BS) and the Chinese Hypoglycemia Fear-Worry Scale (CHFS-II-WS), comprising 15 and 18 items, respectively. It assesses patients’ fear and anxiety related to hypoglycemia experiences, using a 5-point Likert scale ranging from 0 (“never”) to 4 (“always”). Scores span from 0 to 52, with higher scores indicating greater fear of hypoglycemia. The scale’s internal consistency (Cronbach α) is 0.904, with a test-retest reliability of 0.911.

#### Toronto Alexithymia Scale

The Chinese adaptation of the Toronto Alexithymia Scale (TAS-20), translated by Jinyao et al [13], serves as a reliable measure for alexithymia. It features a Cronbach α of 0.83 and a test-retest reliability of 0.87. The scale, encompassing 20 items across 3 dimensions—difficulty identifying feelings, difficulty describing feelings, and externally oriented thinking—is scored on a 5-point scale with a total score range of 20 to 100. A score of 61 or above indicates the presence of alexithymia.

#### Quality Control Measures

Before the commencement of the project, all research personnel underwent standardized training to ensure a consistent understanding and application of the study procedures. To mitigate the risk of information distortion, standardized directives were used alongside a 3-tier data-cleaning methodology for meticulous data collection. Participant selection adhered rigorously to predefined inclusion and exclusion criteria, minimizing selection bias.

To further reduce sampling errors, participants were allocated into groups using a randomized method. Quality control of inspections was ensured through dedicated staff oversight and the regular calibration of instruments. Data integrity was reinforced by dual-entry verification, with 2 individuals independently recording and checking the data. Additionally, 15% of the data set was randomly selected for further validation to uphold data reliability.

Throughout the study, regular team meetings facilitated prompt communication among researchers, essential for the successful execution of the project. The research team consistently monitored treatment outcomes at predetermined intervals, systematically reviewed and verified data, and performed secondary analyses or exclusions as necessary to guarantee data precision.

#### Statistical Analysis Methodology

Data analysis was conducted using the SPSS statistical software (version 26.0; IBM Corp). Categorical data were summarized as frequencies and percentages. Continuous variables, including patients’ FBG, 2-hour postprandial blood glucose, and glycosylated hemoglobin levels, were expressed as mean (SD). The independent sample *t* test was applied for the comparison of continuous variables between 2 groups, while the chi-square test was used for the analysis of categorical data. A *P* value of less than .05 was considered indicative of a statistically significant difference.

## Results

### Assessment Observations

#### Overview

**Figure 1:**
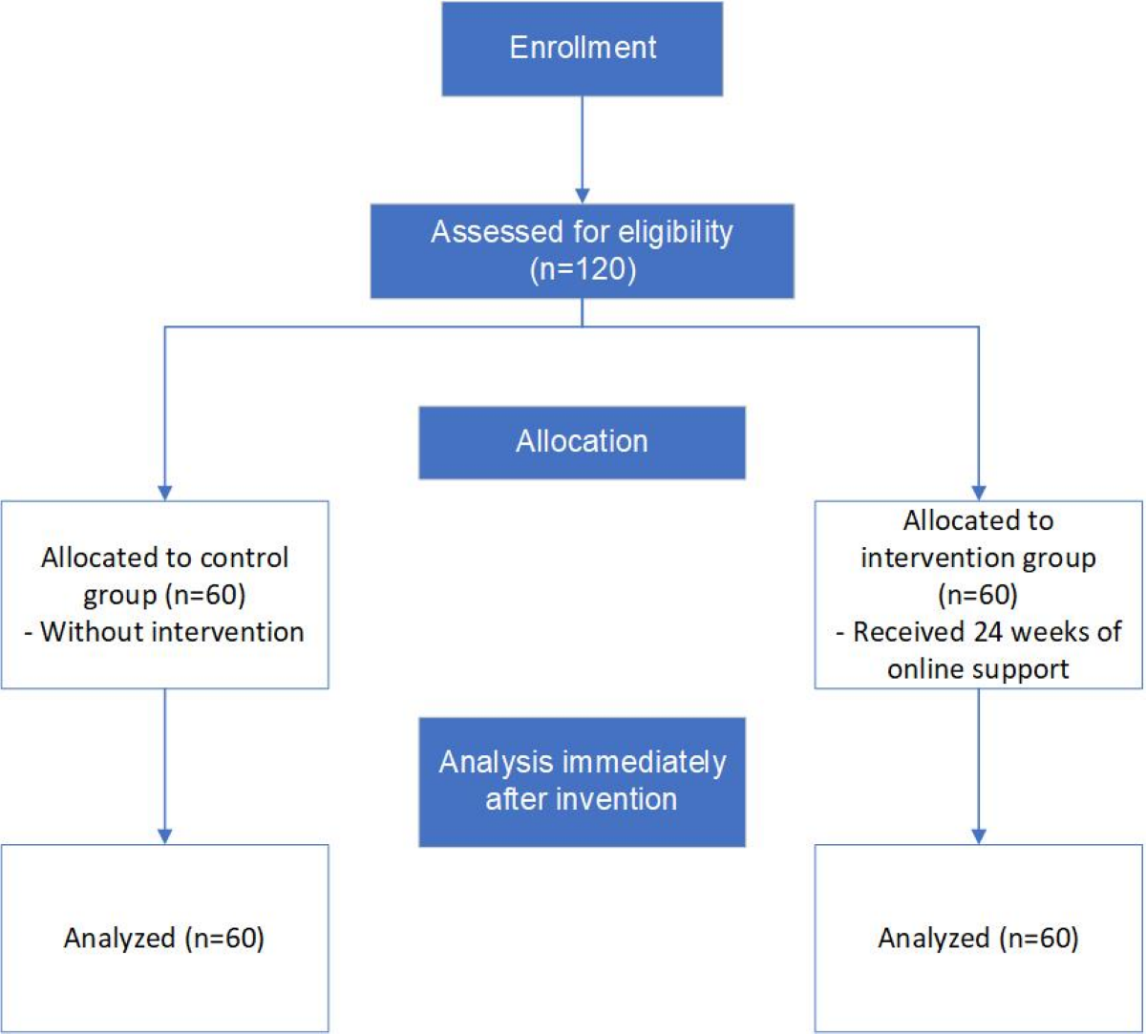
CONSORT (Consolidated Standards for Reporting Trials) diagram of participation flow in the study.

A comprehensive assessment was conducted on 120 newly diagnosed patients with type 2 diabetes at the onset of the intervention, as described in the CONSORT (Consolidated Standards for Reporting Trials) diagram in Figure 1 (Multimedia Appendix 1). This assessment aimed to evaluate the participants’ foundational knowledge of diabetes and their management practices. Baseline data between the 2 groups (each comprising 60 participants) showed no statistically significant difference (*P*>.05), confirming their comparability. Detailed baseline data for both groups are presented in Table 1.

**Table 1:** Comparison of sociodemographic data of the 2 groups of research subjects.

Variables		Control group (n=60)	Online feedback group (n=60)	Statistical value	*P* value
**Sex, n (%)**				0.14^a^	>.05
	Male	36 (60)	34 (57)		
	Female	24 (40)	26 (43)		
Age (years), mean (SD)		45.17 (8.60)	47.88 (11.39)	–1.471 (118)^b^	.93
**Education, n (%)**				1.768^c^	.62
	Primary school and below	6 (10)	3 (5)		
	Junior high school and below	6 (10)	9 (15)		
	High school or secondary school	27 (45)	25 (42)		
	College and above	21 (35)	23 (38)		
**Marital status, n (%)**				0.789^a^	.67
	Unmarried	5 (8)	7 (12)		
	Married	49 (82)	45 (75)		
	Divorced or widowed	6 (10)	8 (13)		
**Payment method for medical expenses, n (%)**				0.976^a^	.61
	Municipal medical insurance	34 (57)	29 (48)		
	District medical insurance	18 (30)	20 (33)		
	Other	8 (13)	11 (18)		
**Labor intensity, n (%)**				0.311^c^	.86
	Mild	20 (33)	29 (37)		
	Moderate	34 (57)	20 (52)		
	Severe	6 (10)	11 (12)		

^a^*c*^2^.

^b^*t* test value.

^c^*Z* value.

#### Fear of Hypoglycemia

A significant proportion of participants (n=86, 72%) reported a fear of hypoglycemia. This fear often stemmed from a lack of understanding about how to manage low blood sugar levels and the potential consequences of hypoglycemia.

#### Misunderstandings About Dietary Requirements

Approximately 78 (65%) participants had misconceptions regarding dietary requirements. These misunderstandings included incorrect beliefs about carbohydrate restrictions, meal timing, and the role of diet in managing blood glucose levels.

#### Poor Adherence to Medication Regimens

About 70 (58%) participants demonstrated poor adherence to their prescribed medication regimens. This nonadherence was largely due to misunderstandings about the effects and importance of their medications, leading to inconsistent usage and suboptimal blood glucose control.

These observations were instrumental in customizing the educational content and intervention strategies for each patient. By addressing specific knowledge gaps and fears, the intervention was able to provide targeted education and support, thereby improving the overall effectiveness of diabetes management for the participants.

Among the participants, 78 (65%) opted to use videos as their primary learning tool, while 42 (35%) chose brochures. The video group demonstrated a higher level of engagement and better retention of information, as evidenced by their active participation in follow-up sessions and accurate recapitulation of disease-related information. Comparatively, the brochure group also showed improvements but at a slightly lower rate. The impact of these tools on glycemic control was significant, with the video group showing a more substantial reduction in FBG and HbA_1c_ levels compared to the brochure group (*P*<.05).

### Comparative Analysis of Pre- and Postintervention Glycemic Indices

Following a 24-week intervention, both patient groups exhibited significant reductions in FBG scores (*P*<.05), with a notably greater decrease observed within the online feedback group (*P*<.05; refer to Table 2). In the online teaching group, pre- and postintervention comparisons revealed statistically significant differences in FBG, 2hPG, and HbA_1c_ scores (*P*<.05). Similar significant changes were observed in the control group for these indices (*P*<.05). Postintervention, the glycemic indices of the online feedback group were significantly lower than those of the control group.

**Table 2:** Comparison of glucose metabolism index scores between the 2 groups before and after intervention.

Group	Count, n	FBG^a^, mean (SD)			*t* test (*df*)		*P* value		2hPG^b^, mean (SD)			*t* test (*df*)		*P* value		HbA_1c_^c^, mean (SD)			*t* test (*df*)		*P* value
		Before intervention^d^	After intervention^e^					Before intervention^f^		After intervention^g^					Before intervention^h^		After intervention^i^				
Control group	60	8.54 (2.32)	6.43 (1.45)	5.974 (59)		<.001		13.64 (4.34)		12.54 (3.35)	1.554 (59)		.12		7.94 (2.92)		7.07 (1.89)	1.934 (59)		.06	
Online feedback group	60	8.39 (2.04)	5.74 (1.66)	7.804 (59)		<.001		13.31 (3.72)		10.88 (2.59)	4.153 (59)		<.001		7.89 (2.84)		6.03 (1.66)	4.38 (59)		<.001	

^a^FBG: fasting blood glucose.

^b^2hPG: 2-hour postprandial glucose.

^c^HbA_1c_: hemoglobin A_1c_.

^d^t_59_=0.376; *P*=.71.

^e^t_59_=2.425; *P*=.02.

^f^t_59_=0.447; *P*=.66.

^g^t_59_=3.037; *P*=.003.

^h^t_59_=0.095; *P*=.92.

^i^t_59_=3.203; *P*=.002.

### Comparative Analysis of Pre- and Postintervention Hypoglycemia Fear Scores

Following a 6-month intervention period, a notable decline in CHFSⅡ-BS and CHFSⅡ-WS scores was observed across both patient groups, with the reduction being significantly greater in the online feedback group (*P*<.05). Furthermore, after the intervention, scores for both CHFSⅡ-BS and CHFSⅡ-WS in the online feedback group were significantly lower than those recorded in the control group (*P*<.05), as detailed in Table 3.

**Table 3:** Comparison of hypoglycemic fear scores between the 2 groups before and after intervention.

Group	Count, n	CHFSⅡ-BS^a^, mean (SD)		*t* test (*df*)	*P* value	CHFSⅡ-WS^b^, mean (SD)		*t* test (*df*)	*P* value
		Before intervention^c^	After intervention^d^			Before intervention^e^	After intervention^f^		
Control group	60	24.53 (5.62)	20.43 (4.15)	4.546 (59)	<.001	18.23 (5.24)	16.51 (4.12)	1.999 (59)	.048
Online feedback group	60	23.29 (6.24)	17.54 (3.97)	6.022 (59)	<.001	19.34 (5.36)	12.39 (4.59)	7.629 (59)	<.001

^a^CHFS-II-BS: Chinese Hypoglycemia Fear-Behavior Scale.

^b^CHFS-II-WS: Chinese Hypoglycemia Fear-Worry Scale.

^c^t_59_=1.144; *P*=.25.

^d^t_59_=3.898; *P*<.001.

^e^t_59_=–1.147; *P*=.25.

^f^t_59_=5.174; *P*<.001.

### Comparative Analysis of Pre- and Postintervention Alexithymia Levels

Before the intervention, baseline alexithymia levels were comparable between the groups. Post intervention, comparative analysis revealed a significant reduction in TAS-20 scores within both cohorts, indicative of decreased alexithymia levels. Notably, the reduction was more pronounced in the group receiving the specialized intervention (*P*<.05), compared to the control group (Table 4).

**Table 4:** Comparison of levels of affective disorder between the 2 groups before and after intervention.

Group	Count, n	TAS-20^a^, mean (SD)		*t* test (*df*)	*P* value
		Before intervention^b^	After intervention^c^		
Control group	60	60.54 (9.72)	57.43 (7.55)	1.953 (59)	.05
Online feedback group	60	59.19 (10.04)	51.54 (6.67)	4.916 (59)	<.001

^a^TAS-20: Toronto Alexithymia Scale.

^b^t_59_=0.748; *P*=.46.

^c^t_59_=–4.529; *P*<.001.

## Discussion

### Impact of Comprehensive Assessment

The detailed baseline assessment provided a nuanced understanding of the patient’s initial conditions and challenges. The high prevalence of hypoglycemia fear and misconceptions about diabetes management underscored the necessity for targeted educational interventions. By identifying these specific needs early, the intervention could be more precisely tailored to address the gaps in knowledge and self-management skills, thereby enhancing the overall effectiveness of the program.

### Effectiveness of Educational Tools

The study found that videos were more effective than brochures in enhancing patient engagement and knowledge retention. Patients who used videos showed a greater reduction in glycemic indices and better comprehension of diabetes management principles. This suggests that video-based education may be a more powerful tool for patient education in diabetes management compared to traditional brochures.

### Advancing Diabetes Management Through Information Technology: a Superior Alternative to Traditional Models

Diabetes management necessitates comprehensive care strategies and meticulous glycemic control. Patients increasingly seek personalized, home-based guidance from health care professionals, underscoring the pivotal role of home and community settings in rehabilitation efforts [14,15]. Our preliminary research revealed a significant demand for home-based care among patients with diabetes, paralleling the findings of Simsek et al [16] regarding chronic disease management in the older population. This “sinking” care model, designed to address both physical and psychological needs, faces challenges due to slow development, service uniformity, process inconsistencies, and personnel shortages, struggling to meet the escalating care demands [17].

Professor Sika of the Mayo School of Medicine emphasizes the current period as an optimal time to leverage “Internet+” technologies in the home health and care of older people, driven by both national policies and societal needs [18]. The era of big data heralds a paradigm shift in health management, characterized by comprehensive interventions, online consultations, risk identification, and chronic disease management, facilitated by full-cycle coverage, precision, wide reach, and rapid feedback. The integration of health monitoring systems and medical data platforms enables dynamic patient monitoring and personalized guidance, bridging the gap between health care providers and patients [19].

Community health service agencies serve as primary gateways for chronic disease management, with online platforms enhancing coordination, organization, and multidimensional monitoring. This model addresses the challenges of professionalism in remote health management and fosters resource sharing, interoperability, and effective integration of health technical personnel. For patients with newly diagnosed type 2 diabetes, digital platforms and information systems offer tailored health management guidance, marking a significant improvement in patient care.

### Enhancing Glycemic Control in Newly Diagnosed Type 2 Diabetes Mellitus Through Online Feedback: an Application of King’s Goal Attainment Theory

The study’s findings reveal that using an online teach-back method, underpinned by King’s Goal Attainment Theory, leads to significant improvements in glycemic control among patients with first-onset type 2 diabetes mellitus (T2DM). Notably, FBG, 2hPG, and HbA_1c_ levels exhibited a consistent decline across both study groups, with the online feedback group showing superior outcomes. This suggests that the online feedback approach not only enhances patients’ glucose metabolism indices but also contributes to an improved quality of life and reduced risk of diabetes-related complications. Despite these positive trends, the control group did not demonstrate statistically significant changes in 2hPG and HbA_1c_ levels (*P*>.05), potentially attributable to the limited duration of observation and the inherently slow response of glycemic indicators.

The online feedback method, aligned with King’s theory, leverages recent dietary records to offer personalized nutritional guidance, promoting healthier dietary choices, regular physical activity, and mood regulation. Such comprehensive support underscores the method’s efficacy in aiding patients to meet glycemic targets. Supporting this, the study by Celik et al [20] on diabetes education among hospitalized patients with type 2 diabetes in Turkey found that repeated, multimodal diabetes education significantly improved self-care and glycemic control (*P*<.05), mirroring the outcomes observed in our investigation.

King’s Goal Attainment Theory, central to our intervention, champions a patient-centered approach, emphasizing active patient engagement in health management through ongoing interactive communication with health care providers. This dynamic process fosters a collaborative effort toward achieving rehabilitative goals, thereby enhancing patients’ understanding of their condition and adherence to treatment regimens outside the hospital setting. Recognizing the varied needs and motivations across different stages of behavioral change, our approach advocates for tailored interventions to support the development of healthier behaviors effectively.

Given the characteristics of type 2 diabetes, including short-term hospitalizations, long-term outpatient care, and the need for effective community management, a tripartite model encompassing hospital, community, and family interventions is proposed. This model aims for comprehensive monitoring and follow-up, ensuring a cohesive support system for patients across various care settings.

### Impact of Online Teach-Back Method on Psychological Control in Newly Diagnosed T2DM

Diabetic alexithymia and fear represent prevalent adverse psychological responses impacting patients with diabetes, often undermining self-management practices and overall quality of life [21]. Despite their significance, these emotional challenges are seldom recognized and addressed within clinical settings. This study uses a structured 6-step intervention mapping process—encompassing a logical model of the problem, program outcomes and objectives, design, production, execution plan, and evaluation strategy [22]. This methodology is grounded in educational and psychological frameworks, notably Bloom’s Taxonomy of Educational Objectives and Bandura’s Social Cognitive Theory, aiming to bolster psychological regulation among patients.

Our findings indicate that patients with T2DM exhibit moderate levels of alexithymia, aligning with the observations of Celik et al [23]. Postintervention assessments revealed significant improvements in the online feedback group, with notable reductions in the scores of the CHFSⅡ-WS, CHFSⅡ-BS, and TAS-20, compared to the control group (*P*<.05). These outcomes underscore the efficacy of the online teach-back approach, informed by King’s Goal Attainment Theory, in enhancing coping mechanisms and life quality for patients with initial T2DM.

This study highlights the pivotal role of King’s theory in addressing the dual challenges of limited medical resources and patients’ diminished self-awareness, which often impede effective diabetes self-management. By leveraging this theoretical framework, our intervention promotes the dissemination of essential disease information and health education resources, fostering a patient-centered, interactive communication model. The observed significant amelioration in alexithymia and hypoglycemia fear levels substantiates the model’s potential in elevating psychological well-being and control among patients with diabetes, thereby affirming the transformative impact of integrating high-quality, theory-based online feedback mechanisms in diabetes care.

### Limitations

First, the constrained sample size poses challenges to the analytical precision, underscoring the necessity for larger cohorts to validate findings and examine nuanced changes in T2DM-specific health indicators. Second, there exists an imperative for future research to establish localized, comprehensive health management models that encompass a broader spectrum of chronic conditions, thereby facilitating a more holistic approach to patient care. Finally, the potential influence of extraneous social factors, including occupational status and comorbid conditions, on the study outcomes cannot be overlooked. Future investigations should aim to meticulously refine inclusion and exclusion criteria to mitigate such variables’ impact.

### Conclusions

The application of the online teach-back method, grounded in King’s Goal Attainment Theory, emphasizes dynamic online communication. This approach transitions from leveraging the potential of intervention strategies to fostering patients’ subjective initiative and internal motivation for behavioral change. Such a methodological shift holds substantial promise for enhancing health outcomes in patients with newly diagnosed T2DM.

## Appendix

Multimedia Appendix 1 CONSORT (Consolidated Standards for Reporting Trials) checklist.

## Abbreviations

2hPG: 2-hour postprandial glucose
CHFS-II: Chinese Version of the Hypoglycemia Fear Survey II
CHFS-II-BS: Chinese Hypoglycemia Fear-Behavior Scale
CHFS-II-WS: Chinese Hypoglycemia Fear-Worry Scale
CONSORT: Consolidated Standards for Reporting Trials
FBG: fasting blood glucose
HbA1c: hemoglobin A1c
MMSE: Mini-Mental State Scale
T2DM: type 2 diabetes mellitus
TAS-20: Toronto Alexithymia Scale
